# Septic cardiomyopathy or myocardial infarction?: A case report of septic shock with ST-segment elevation on ECG

**DOI:** 10.1097/MD.0000000000041454

**Published:** 2025-01-31

**Authors:** Haolei Gao, Xiaodong Wang, Qingyue Yang

**Affiliations:** a Department of Anesthesiology, Weihai Municipal Hospital, Cheeloo College of Medicine, Shandong University, Weihai City, Shandong Province, China; b Department of Anesthesiology, Weihai Municipal Hospital, Cheeloo College of Medicine, Shandong University, Weihai City, Shandong Province, China; c Department of Critical Care Medicine, Laizhou People’s Hospital, Yantai City, Shandong Province, China.

**Keywords:** myocardial infarction, sepsis, sepsis shock, septic cardiomyopathy

## Abstract

**Rationale:**

Sepsis is one of the most prevalent and deadly diseases today. Sepsis involving the heart can progress to septic cardiomyopathy; however, there is a lack of uniform diagnostic criteria. A review of the literature reveals a paucity of literature on sepsis combined with acute myocardial infarction (AMI) and no reports on emergency surgical treatment.

**Patient’s concerns:**

A 52-year-old patient with trauma-induced sepsis leading to acute heart failure with elevated ST-segment on electrocardiogram and postoperative coronary angiography suggestive of AMI.

**Diagnoses:**

Small bowel rupture, infectious shock, AMI, hypertensive disease, old cerebral infarction.

**Interventions:**

The patient was admitted to the hospital and immediately underwent emergency surgery to remove the infected focus, with treatment with meropenem for anti-infection, ambroxol for sputum, parenteral nutritional support, sedation and analgesia, esmolol to control the ventricular rate, uradil to control blood pressure, and transfusion of red blood cells and plasma for correction of anemia and coagulation functions. Coronary angiography was performed 6 months later.

**Outcomes:**

The patient was discharged after showing signs of improvement and was subsequently monitored in an outpatient clinic setting. At the time of writing, the patient is still alive and well.

**Lessons:**

In cases of acute heart failure resulting from trauma-induced sepsis, it is crucial to consider myocardial ischemia as a potential factor. Early surgical removal of infected foci may prove beneficial in improving the patient’s prognosis. However, differentiating between septic cardiomyopathy and sepsis-combined myocardial infarction can be challenging, and the appropriateness of the diagnostic criteria for sepsis at this stage is debatable.

## 1. Introduction

Sepsis is defined as a dysregulated host response to infection that leads to life-threatening organ dysfunction.^[1]^ It can affect cardiac function and lead to septic cardiomyopathy. According to a recent report on the Global Burden of Diseases, there were an estimated 48.9 million cases of sepsis and 11.0 million sepsis-related deaths worldwide in 2017. This represents 19% of all global deaths.^[1]^ Septic cardiomyopathy is a condition often characterized by temporary and reversible changes in cardiac function. This sepsis-induced acute cardiac dysfunction is unrelated to ischemia. This case report describes a patient with septic shock caused by abdominal injury. The patient exhibited ST-segment elevation in multiple leads on the electrocardiogram (ECG). Coronary CT angiography performed 7 months post-surgery also indicated a diagnosis of myocardial infarction (MI). The literature on patients with sepsis complicated by acute MI (AMI) is limited. This study reviews relevant literature and discusses the relationship between sepsis and AMI. The aim is to provide insight into the pathobiology, diagnostic approaches, and treatment options for this condition to guide clinical decision-making.

## 2. Case presentation

**Figure 1.**
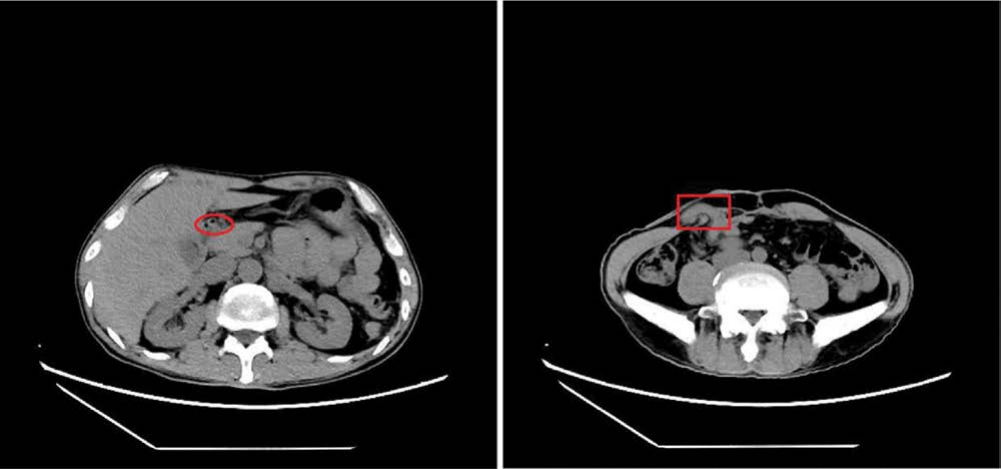
Abdominal CT findings. Red circle: intra-abdominal free gas. Red rectangle: small intestine overflowing the abdominal wall.

**Figure 2.**
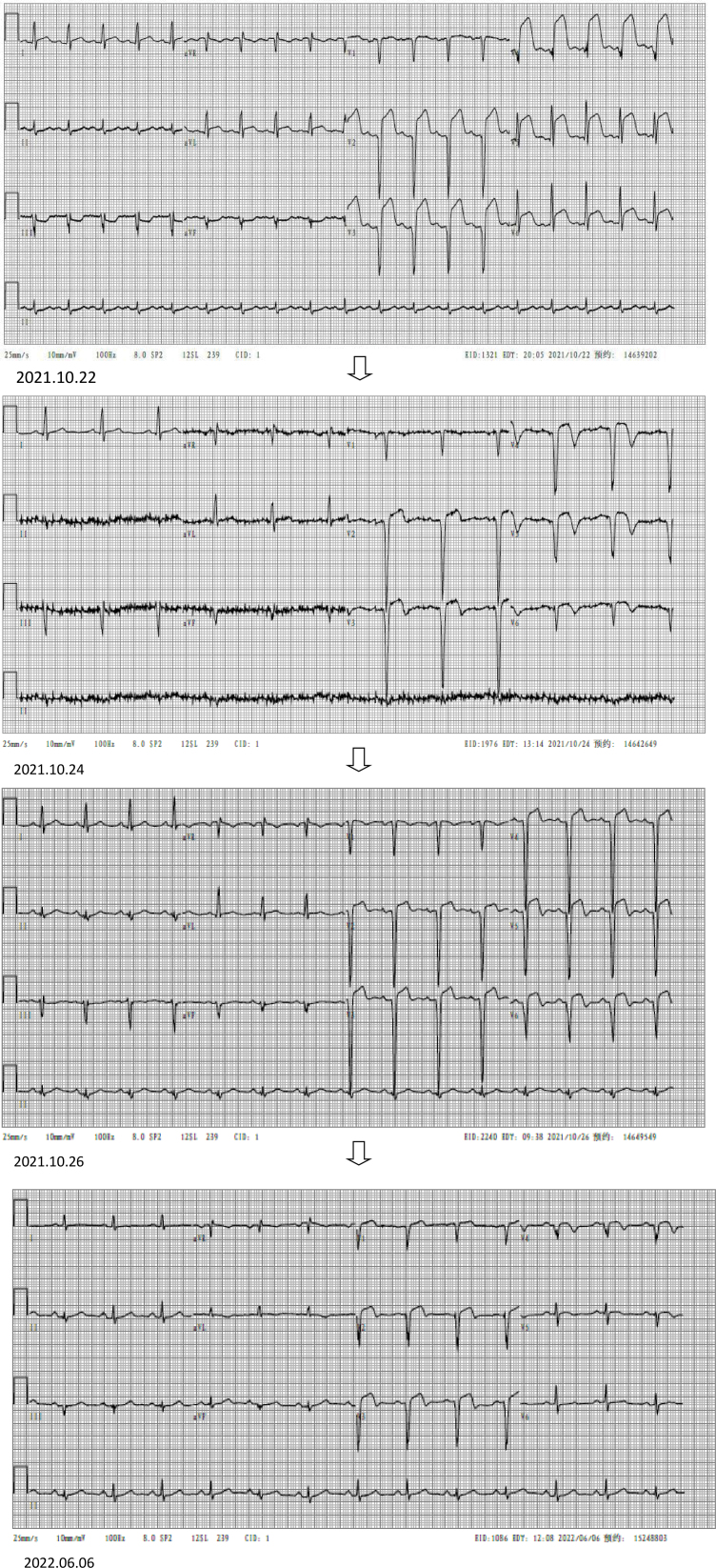
Evolution of the ECG. ECG = electrocardiogram.

The patient, a 52-year-old man, had a 3-year history of cerebral infarction but had recovered well with conservative treatment with no subsequent lingering effects. He also had a 1-year history of untreated hypertension. No prior issues with cardiovascular disease, chest tightness, chest pain, or other symptoms were reported. He received a blow to his back from a piece of wood while performing physical labor the day before, which had compressed his lower abdomen against the stairs. This incident caused unbearable lower abdominal pain without external abdominal wall injuries. After returning home to rest, the patient experienced persistent and worsening pain, leading to hospitalization for treatment on October 22, 2021. Physical examination upon admission indicated apathy, cold limbs, blood pressure of 87/62 mm Hg, pulse rate of 108 beats/min, respiratory rate of 27 breaths/min, abdominal tension, lower abdominal tenderness and rebound pain, and tympanic sounds on percussion. Abdominal CT revealed multiple intestinal changes, peritoneal effusion, hematogenous accumulation, some free gas in the peritoneal cavity, and possible intestinal rupture (Fig. 1). The laboratory results were as follows: BNP: 5170.40 pg/mL (reference range 300–900); Troponin l: 1.29 ng/mL (reference range < 0.14); CK: 369.6 U/L (reference range 35–190); CK-MB: 38.1 U/L (reference range 0–25); WBC: 17.77 × 10^9^/L (reference range 3. 5–9.5); CRP: 237.07 mg/L (reference range 0–3); HGB: 157 g/L (reference range 130–175); PLT: 209 × 10^9^/L (reference range 125–350); creatinine: 249.9 µmol/L; BUN: 77.9/L (reference range 3–17). The ECG suggested sinus tachycardia and acute extensive front-wall MI (Fig. 2). Cardiac ultrasound showed nonobstructive hypertrophic myocardial disease, increased left room, minor valve reflux, and left heart relaxation function loss. The levels of inflammatory parameters, including serum bilirubin, creatinine, and myocardial enzymes, were increased (Table 1). The patient’s laboratory findings and CT scan results indicated possible intestinal rupture, causing purulent disease and multi-organ damage. Considering the patient’s critical condition, a cesarean section was recommended to control the infection and aid the patient’s recovery. Therefore, an emergency surgery was scheduled after multi-disciplinary discussions.

**Table 1:** Infection and cardiac function-related hematological and investigation data.

	Reference	Day 1	Day 2	Day 3	Day 4	Day 5	Day 6	Day 7	Day 8	Day 9	Day 10	Day 11	7 month
WBC (×10^3^/μL)	3.5–9.5		9.68			11.36		18.7		18.13		17.12	6.66
RCRP (mg/L)	0–3		7.901	8.323	13.3	15.66		13.88		12.19		8.24	
NT-proBNP (pg/mL)	300–900	5170.4				2177		1513		621.5			
HS-TnT (pg/mL)	0–14		574.7	1431	1319	1648		2314		1700		1082	
CK (U/L)	35–190	369.6	1616.5	1153.8	496.8	325.7		208.9		104.9		101.8	66.9
CK-MB (U/L)	0–25	38.1	127	51.6	33.4	18.9		15		13		11.9	
Troponinl (ng/mL)	<0.14	1.29											
Lac (mMol/L)	0.5–1.8	4	1.8	1	0.8	1				1.2			
PCT (ng/mL)	0–0.046					12.1		1.42		0.69		0.44	
EF	(50–70)%	58	47					50					64
HR	(60–100)	117	95	86	83	92	85	80	80	80	76	77	75
ICON	(37.5–62.5)	24.1	19.9	30.7									

BNP = brain natriuretic peptide, BUN = blood urea nitrogen, CK = creatine kinase, CK-MB = creatine kinase-muscle/brain, EF = ejection fraction, HGB = hemoglobin, HR = heart rate, HS-TNT = high-sensitivity troponin T, ICON = impedance cardiography, Lac = lactic acid, NT-proBNP = N-terminal pro-brain natriuretic peptide, PCT = procalcitonin, PLT = platelets, RCRP = C-reactive protein, WBC = white blood cell.

Following general anesthesia induction, the patient had a ventricular rate of 127 beats/min and a blood pressure of 77/45 mm Hg. Norepinephrine was administered intravenously to achieve hemodynamic stability, while anesthesia was maintained with sevoflurane inhalation and remifentanil intravenous infusion. Surgical exploration of the abdomen revealed approximately 1000 mL of purulent and bloody fluid within the pelvic cavity. The liver, gallbladder, stomach, spleen, and rectum appeared normal. Necrosis was noted in a segment of the small intestine approximately 6 cm in length and 80 cm proximal to the ileocecal junction. A 3-cm perforation was visible in the middle, spilling intestinal contents. A 4-cm tear was also found along the mesenteric border. Large amounts of formed stool were found in the small intestine and colon. The patient underwent abdominal exploration, partial small bowel resection, double-barrel ileostomy at the terminal ileum, and pelvic abscess drainage. The mean arterial pressure remained below 65 mm Hg during the surgery, even after infusion of 1500 mL crystalloid and 500 mL colloid. Subsequently, 870 mL of plasma and 2 units of red blood cells were transfused to stabilize the hemodynamics, leading to the gradual norepinephrine discontinuation. During the surgery, the patient suffered a 1000 mL blood loss and had a urine output of 50 mL. After the procedure, the patient was placed on a breathing tube and was immediately transferred to the ICU for further treatment. The treatment included meropenem for infection, ambroxol for sputum, enteral nutrition support, sedatives, analgesic infusion, esmolol for heart rate control, urapidil for blood pressure control, and transfusion of red blood cells and plasma to correct anemia and improve coagulation function. The patient’s cardiac enzymes and ECG changes were monitored continuously. The patient’s vital signs stabilized after 3 days, and he was transferred to the general ward of the gastrointestinal surgery department. The cardiology department recommended coronary computed tomography angiography (CTA) for further treatment, but the patient refused. The patient improved and was discharged on November 2, 2021.

## 3. Follow-up

**Figure 3.**
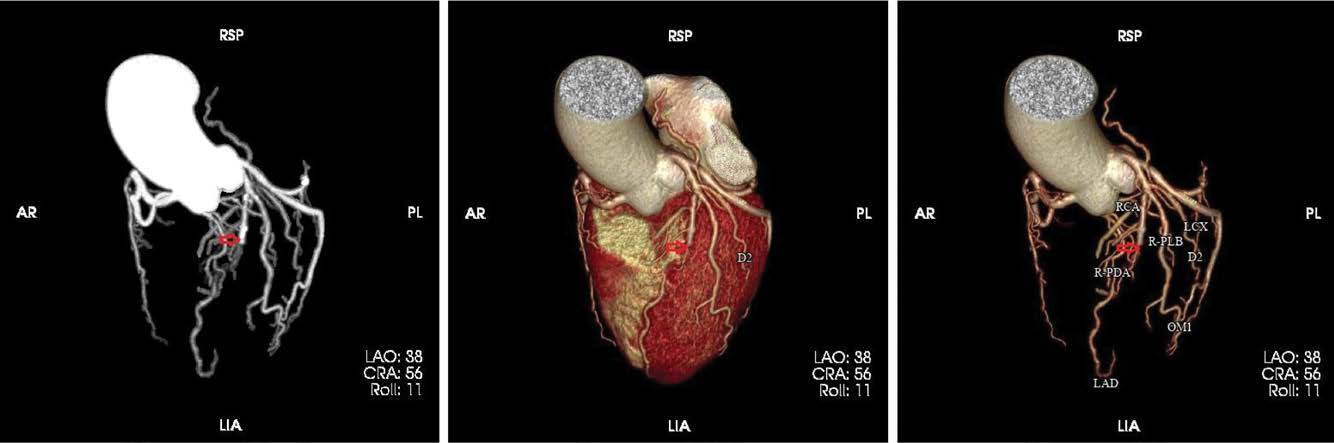
Coronary CTA. The red arrow points to an occlusion of the middle portion of the anterior descending coronary artery. CTA = computed tomography angiography.

**Figure 4.**
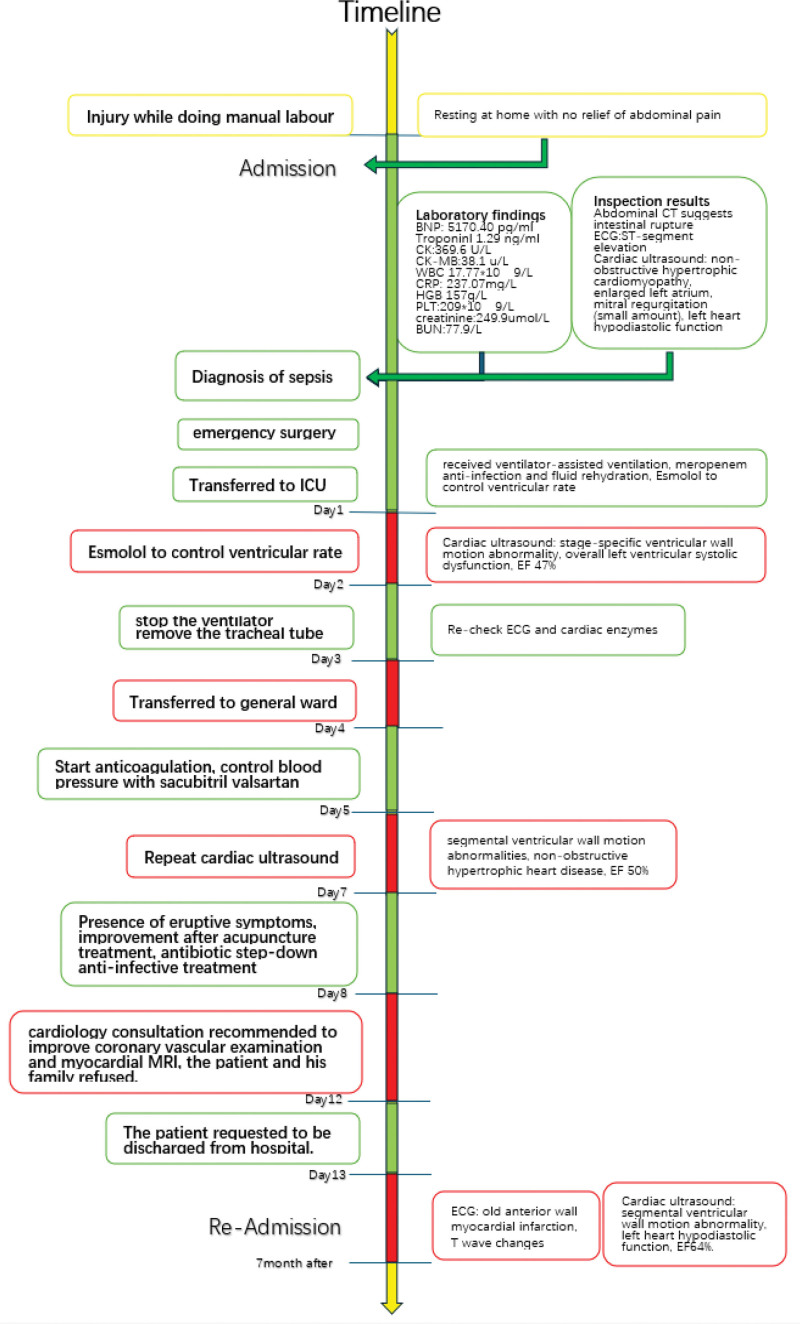
Timeline. BNP = brain natriuretic peptide, BUN = blood urea nitrogen, CK = creatine kinase, CK-MB = creatine kinase-muscle/brain, CRP = C-reactive protein, HGB = hemoglobin, PLT = platelets, WBC = white blood cell.

After discharge from the hospital, the patient recuperated at home. He was readmitted to the hospital for ostomy reconstruction on June 6, 2022. The patient did not recall a history of MI, chest pain, breathlessness, or other discomforts. Additionally, he could tolerate intense physical activity. Specialist examination revealed a heart rate of 66 bpm and muffled heart sounds without murmurs in the auscultation area of each valve. The patient then underwent a cardiac ultrasound, ECG, and coronary CTA (Fig. 3). The ECG (Fig. 2) revealed an old anterior MI, while a cardiac ultrasound showed abnormal ventricular wall motion and a left ventricular ejection fraction of 64%. Additionally, coronary CTA indicated coronary atherosclerotic heart disease and a mid-segmental occlusion of the anterior descending artery. An old MI was diagnosed, together with coronary atherosclerotic heart disease. The patient and his family were informed that coronary angiography was necessary to confirm the diagnosis and initiate interventional treatment, where required, and that after the interventional treatment, he would be placed on dual antiplatelet drugs for at least 6 months to 1 year, which could affect the timing of surgery. The patient and his family expressed their informed understanding but refused interventional treatment, opting instead for conservative treatment with drugs. The patient requested discharge and is currently thriving. This paper will show the development through a timeline (Fig. 4) so that the reader can more clearly understand the chronology of the relevant events and their interrelationships.

## 4. Discussion

The patient had cardiac dysfunction upon admission. How do we define this cardiac dysfunction?

The patient’s diagnosis of sepsis due to traumatic bowel rupture is clear. A sepsis-induced systemic inflammatory response can lead to multi-organ dysfunction. Cardiac involvement can cause impaired cardiac function, known as septic cardiomyopathy, a common and serious complication in patients with sepsis. At the time of admission, we strongly suspected septic cardiomyopathy. However, there were no clear diagnostic criteria for septic cardiomyopathy.

Septic cardiomyopathy is characterized by temporary and reversible acute ventricular dysfunction with impaired contractility that usually resolves within 7 to 10 days.^[2]^ The currently more accepted criteria are: (1) ventricular dilation with increased ventricular compliance and normal to low filling pressure, different from the typical cardiogenic shock pattern with elevated ventricular pressures; (2) decreased ejection fraction without a corresponding decrease in stroke volume; (3) diminished response to fluid resuscitation and catecholamines; (4) resolution within 7 to 10 days; (5) exclusion of acute coronary syndrome as etiology.^[3]^ Several underlying mechanisms have been suggested, including inflammation, calcium handling, mitochondrial dysfunction, complements, exosomes, and ncRNAs, and are still being explored.^[4]^

The diagnosis of septic shock in this report was established based on international standardized diagnostic criteria^[5]^ along with the patient’s medical history, imaging examinations, and laboratory test results. Although the patient had no previous history of heart disease, he exhibited cardiac dysfunction, including ECG changes, elevated cardiac enzyme levels, and decreased left ventricular ejection fraction during the septic shock. The patient’s cardiac function improved gradually after 12 days of treatment, and the prognosis was good. These symptoms closely resemble septic cardiomyopathy, suggesting that the patient may have developed this condition. However, the patient’s coronary CTA results after readmission to the hospital revealed coronary atherosclerotic disease and mid-segmental occlusion of the anterior descending artery which did not align with the currently more accepted diagnostic criteria.

MI is currently indicated by changes in cardiac troponin concentrations with at least 1 value above the 99th percentile upper reference limit accompanied by features of acute myocardial ischemia with at least one of the following: symptoms of acute myocardial ischemia, ECG, noninvasive imaging, or invasive coronary angiography.^[6]^ Based on these criteria, combined with the patient’s troponin level and coronary CTA results, the diagnosis of acute MI was clear.

Is it reasonable to diagnose MI in this patient where heart failure was due to infection? Is there an association between sepsis and MI?

Septic cardiomyopathy refers to a sepsis-associated acute cardiac dysfunction syndrome that is unrelated to ischemia in etiology. However, it is important to exclude coronary syndromes as a confounding factor. In this case, the patient had no previous history of coronary syndrome and was engaged in prolonged physical labor with no clinical symptoms before admission. Despite this, the diagnosis of acute MI was clear from the ECG, cardiac enzyme levels, cardiac ultrasound, and late coronary CTA results. This raises the question of whether the patient’s acute MI and sepsis co-occurrence was a chance event or whether they were associated. We reviewed the relevant literature and confirmed that associations between sepsis and MI have been reported. Four major online databases, PubMed, Embase, Web of Science, and Cochrane, were searched to identify studies that met the eligibility criteria. The last date for data retrieval was October 3, 2023, and there were no language restrictions during the search. To improve completeness and prevent omissions, the reference lists of full-text articles were searched manually. The literature review confirmed the association between sepsis and MI.

Allou et al identified concomitant acute myocardial infarction in 78 (5.5%) of 1418 patients hospitalized for severe sepsis who underwent coronary angiography,^[7]^ while Nathan et al reported a case of neonatal enteroviral sepsis combined with ischemic cardiomyopathy,^[8]^ Singh et al reported acute infarction due to septic embolism in methicillin-resistant *Staphylococcus aureus* bacteremia,^[9]^ and Musher et al suggested a causal relationship between acute infection and increased risk of MI.^[10]^

The potential mechanisms underlying infection-induced MI include:

(1) The presence of atherosclerotic plaques containing inflammatory cells and infections elsewhere in the body results in the release of inflammatory cytokines into the circulation. These cytokines can enhance the activity of the inflammatory cells within the plaques and, in turn, can lead to upregulation of host response proteins and promote oxidative bursts, leading to plaque instability.^[11]^
(2) Increased risk of coronary thrombosis at sites of plaque disruption due to enhanced prothrombotic and procoagulant states associated with acute infection.^[12]^
(3) Infection-induced cytokine storms can also damage cardiomyocytes by inhibiting oxygen utilization by the mitochondria, potentially contributing to triggering MI.^[13]^

This case provides insight into the possible reasons for the patient’s good prognosis. For example, MI should be suspected when septic patients present with acute ST-segment elevation on the ECG. If septic shock and acute infarction co-occur, prioritizing the removal of the infected focus may be more beneficial to the patient. Finally, there is a causal relationship between sepsis and MI. However, the previously accepted diagnosis of septic cardiomyopathy is questionable, as the current diagnostic criteria tend to exclude cardiac dysfunction due to myocardial ischemia.

This case highlights the interaction of multiple mechanisms contributing to MI in the context of sepsis. The sepsis-induced systemic inflammatory response, characterized by elevated cytokine levels, likely destabilized preexisting atherosclerotic plaques, while the increased myocardial oxygen demand from septicemia further exacerbated ischemic injury.^[14]^ These mechanisms are consistent with prior studies highlighting inflammatory cytokines and procoagulant states as triggers for plaque rupture and coronary thrombosis during acute infections. Differentiating between sepsis-triggered MI and septic cardiomyopathy remains a significant challenge, as both conditions may present with overlapping features, including elevated cardiac enzymes and ST-segment changes on ECG. In this case, coronary CTA confirming mid-segmental occlusion of the left anterior descending artery supported the diagnosis of MI, despite the initial suspicion of septic cardiomyopathy. The coexistence of sepsis and MI in this patient highlights the need for a comprehensive approach to treatment. While early surgical removal of infected foci is critical for managing sepsis, recognition of ischemic contributions to cardiac dysfunction may prompt timely initiation of medical therapies for MI. This dual-pathway understanding emphasizes the importance of integrating infection control with cardiovascular management in similar cases. Although the diagnosis of MI was supported by imaging and laboratory findings, the inability to perform coronary angiography at the time of the initial event could limit our ability to definitively exclude preexisting coronary artery disease or determine the precise contribution of sepsis-induced mechanisms. The missing baseline cardiac data could introduce uncertainty into differentiating between septic cardiomyopathy and sepsis-associated MI. Hypothetically, if the patient had preexisting coronary artery disease, the diagnosis of septic cardiomyopathy would become less likely, and ischemic mechanisms would take precedence. Conversely, if no prior coronary abnormalities were present, the findings would further substantiate a causal relationship between sepsis and acute MI. This highlights the need for prospective studies to evaluate preexisting cardiac function and imaging to better delineate these conditions.

## 5. Research limitations

The patient’s posthospitalization electrocardiogram results, laboratory findings, cardiac ultrasound, and coronary CTA results 7 months later all suggested the presence of MI. However, although the patient reported no previous history of coronary artery disease or related symptoms, such as chest tightness and chest pain, no preadmission cardiac imaging or diagnostic data, such as baseline electrocardiograms, echocardiograms, or coronary angiography, were available to reflect the cardiac function before the current episode. This limitation prevents a definitive exclusion of preexisting coronary artery disease, introducing uncertainty into the interpretation of whether the MI was entirely sepsis-induced or the result of an underlying chronic condition exacerbated by sepsis. Hypothetically, if preexisting coronary artery disease was present, the interaction between chronic atherosclerosis and acute sepsis-induced inflammatory responses could synergistically contribute to plaque instability and subsequent MI. Furthermore, the lack of coronary angiography at the time of acute illness limits the ability to confirm plaque rupture or thrombosis, which are critical for differentiating septic cardiomyopathy from acute MI. This uncertainty highlights the importance of integrating advanced diagnostic tools, such as coronary angiography or cardiac magnetic resonance imaging, in similar future cases to delineate the underlying pathophysiological mechanisms more precisely. To address this gap, sensitivity analysis will be employed in future studies. For instance, stratifying patients based on their baseline cardiovascular risk factors and assessing outcomes with and without the assumption of preexisting coronary disease may enhance the robustness of the conclusions. Additionally, incorporating serial biomarker assessments, such as high-sensitivity cardiac troponins and inflammatory markers, could help clarify the temporal relationship between sepsis and myocardial ischemia. While the absence of preadmission baseline data, such as ECG, cardiac ultrasound, or coronary imaging limits definitive conclusions regarding preexisting coronary atherosclerosis or myocardial ischemia, a hypothetical scenario can be considered. If such data had indicated significant coronary pathology prior to admission, the interpretation of cardiac dysfunction in this case might shift towards an acute exacerbation of chronic ischemic heart disease due to sepsis. Conversely, a lack of preexisting coronary abnormalities would strongly support the association between sepsis and the acute myocardial injury observed. These hypothetical considerations underline the necessity of future studies to collect and analyze comprehensive premorbid cardiac data to refine diagnostic accuracy and prognostic assessments in similar cases.

## 6. Conclusion

We report the case of a patient with septic shock whose ECG on hospital admission showed multi-lead ST-segment elevation, impaired cardiac function, increased cardiac enzyme levels, and hemodynamic instability. Emergency surgical intervention was performed immediately upon admission to remove the infected foci. Anti-infective therapy was initiated, and the patient made a good recovery following surgery. A follow-up coronary CTA 7 months later revealed a staged occlusion of the middle anterior descending branch without any current obvious sequelae. Furthermore, the patient’s daily activities were not limited. The relevant literature on septic cardiomyopathy, its diagnosis, pathogenesis, and treatment options were reviewed and this information is presented together with the patient’s case summary. The patient did not undergo coronary angiography and coronary stenting even while experiencing concomitant MI. However, the early performance of an etiologically-specific infection foci debridement procedure may have contributed to the favorable postoperative recovery. The exclusion of coronary ischemia from the diagnosis criteria for septic cardiomyopathy is also questioned. It is hoped that the information in this case report will advance the future diagnosis and treatment of similar cases.

## Acknowledgments

Many thanks to the patient for agreeing to post his information. We are also grateful to all the medical staff of Weihai Municipal Hospital who were involved in saving this patient’s life.

## Author contributions

**Conceptualization:** Haolei Gao, Qingyue Yang.

**Data curation:** Haolei Gao, Xiaodong Wang.

**Investigation:** Qingyue Yang.

**Methodology:** Haolei Gao, Qingyue Yang.

**Project administration:** Haolei Gao.

**Supervision:** Haolei Gao.

**Validation:** Haolei Gao.

**Writing – original draft:** Haolei Gao.

**Writing – review & editing:** Haolei Gao, Xiaodong Wang, Qingyue Yang.
